# The Effect of Nitrogen Incorporation on the Optical Properties of Si-Rich a-SiCx Films Deposited by VHF PECVD

**DOI:** 10.3390/mi12060637

**Published:** 2021-05-30

**Authors:** Hongliang Li, Zewen Lin, Yanqing Guo, Jie Song, Rui Huang, Zhenxu Lin

**Affiliations:** School of Materials Science and Engineering, Hanshan Normal University, Chaozhou 521041, China; lihl4@hstc.edu.cn (H.L.); yqguo126@126.com (Y.G.); songjie@hstc.edu.cn (J.S.); rhuang@hstc.edu.cn (R.H.); lzx2016@hstc.edu.cn (Z.L.)

**Keywords:** photoluminescence, thin films, optical properties, SiCx

## Abstract

The influence of N incorporation on the optical properties of Si-rich a-SiC_x_ films deposited by very high-frequency plasma-enhanced chemical vapor deposition (VHF PECVD) was investigated. The increase in N content in the films was found to cause a remarkable enhancement in photoluminescence (PL). Relative to the sample without N incorporation, the sample incorporated with 33% N showed a 22-fold improvement in PL. As the N content increased, the PL band gradually blueshifted from the near-infrared to the blue region, and the optical bandgap increased from 2.3 eV to 5.0 eV. The enhancement of PL was suggested mainly from the effective passivation of N to the nonradiative recombination centers in the samples. Given the strong PL and wide bandgap of the N incorporated samples, they were used to further design an anti-counterfeiting label.

## 1. Introduction

The motivation to realize monolithic optoelectronic integrated circuits has spurred great efforts to explore efficient Si-based light sources that can operate at room temperature and is compatible with the mainstream complementary metal-oxide-semiconductor technology (Fadaly, Dijkstra et al. 2020) [1,2,3,4,5,6]. Thus far, different techniques such as plasma-enhanced chemical vapor deposition and sputtering associated with appropriate post-annealing processing are being employed to obtain efficient light emission from Si-based materials [7,8,9,10,11,12,13,14,15,16]. Among the investigated materials, SiOx and SiNx systems have been reported to exhibit intense light emission [15,16]. However, the wide bandgap of silicon oxide (~8.5 eV) hinders the effective injection of carriers, and the material is thus unsuitable for the fabrication of stable and efficient electroluminescent devices. Meanwhile, a large number of nonradiative recombination centers in silicon nitride systems increase the difficulty of obtaining efficient electroluminescence [17]. In recent years, amorphous silicon carbide (a-SiCx) films have also attracted much attention because of their low-cost preparation and superior physical and chemical properties, such as strong photoluminescence (PL), high doping efficiency, visible region transparency, and immense potential in the fields of Si-based photoelectric integration, photovoltaic cells, and detectors [18,19]. Thus far, a large number of studies have explored the structural, electrical, and optical properties of a-SiCx films [20]. The experimental results have shown that the properties of a-SiCx films are closely related to their contents of C and Si atoms and the bonding configuration between atoms. For example, the valence band edges and conduction band edges of a-SiCx films are contributed by Si and C atoms. An increase in C content in a-SiCx films is conducive to replacing Si–Si bonds, thereby increasing the density of silicon–carbon bonds and thus effectively widening the bandgaps of the films. The incorporation of different atoms into the materials from the working gas is a well-known process affecting the structure and physical properties of the materials [21,22,23,24]. In the previous work of our research group, by incorporating O atoms into a-SiCx films from the oxygen gas flow with PECVD technique, the influence of O doping on the luminescence characteristics of a-SiCx films was studied, and a three-level model based on the luminescence center of a Si dangling bond was established [25]. The net gain coefficient of the film was observed under ultraviolet pumping. Although efficient PL from SiC-based materials has been investigated, progress is slow. This lag is partly due to the lack of information available to correlate PL with other influence factors.

In this study, we showed the influence of N incorporation on the optical properties of Si-rich a-SiCx films. PL measurements combined with X-ray photoelectron spectroscopy (XPS) analysis revealed that an increase in N content resulted in a remarkable enhancement in PL and switcheed the emitted light from the red region to the blue region. The intense tunable light emission was discussed herein. Given the strong PL and wide bandgap of N incorporated samples, they were used to design an anti-counterfeiting label in this work.

## 2. Experimental Details

Amorphous Si-rich a-SiCx films with a thickness of 300 nm were fabricated by very high-frequency plasma-enhanced chemical vapor deposition (VHF PECVD, Shenyang New Blue Sky Vacuum Technology Co., Ltd., Shenyang, China) technology at a substrate temperature of 250 °C; SiH_4_ and CH_4_ were used as reaction gas sources. The flow rates of SiH_4_ and CH_4_ were set at 2.5 and 5 sccm, respectively. During the preparation phase, the flow rates of NH_3_ were introduced and set as 0, 5, 10, and 15 sccm to investigate the effects of N incorporation on a-SiCx. The room temperature PL and PL decay characteristics of the films were measured with an Edinburgh FLS1000 fluorescence spectrometer. The absorption spectra of the films were obtained with a Shimadzu UV-3600 spectrophotometer. The microstructures of the films were evaluated using the Horiba LabRAM HR Evolution Raman spectrometer. The Si, C, and N contents in the films were determined by XPS (Thermo-VG Scientific ESCALAB 250, Waltham, MA, USA). The bonding structures were examined by a Fourier transform infrared (FTIR) spectrometer.

## 3. Results and Discussion

**Figure 1:**
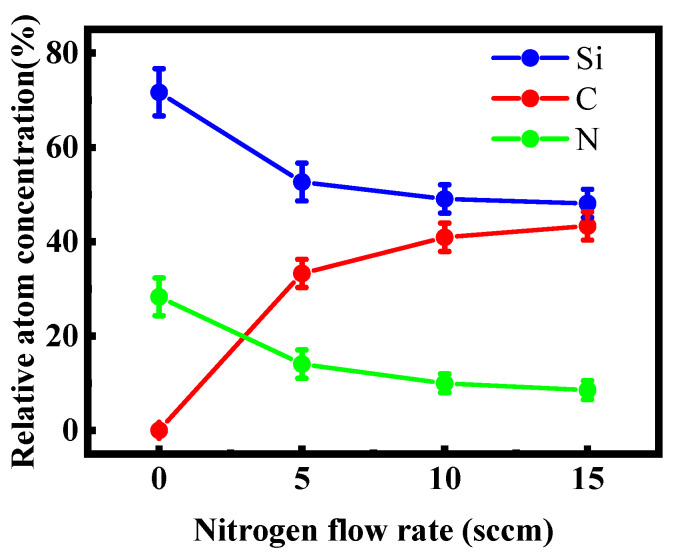
Si, N, and C contents of the samples fabricated at different NH_3_ flow rates.

Figure 1 shows the Si, N, and C contents of the films prepared with different NH_3_ flow rates. When the NH_3_ flow rate was zero, the Si and C contents of the films were 71% and 29%, respectively. These values indicated that the films were Si-rich silicon carbides. As the NH_3_ flow rate increased from 5 sccm to 15 sccm, the film composition changed significantly. When the NH_3_ flow rate was 5 sccm, the Si and C contents of the films remarkably decreased to 53% and 14%, respectively, while the N content rapidly increased to 33%. As the NH3 flow increased further, the Si and C contents of the films decreased continuously while the N content increased.

**Figure 2:**
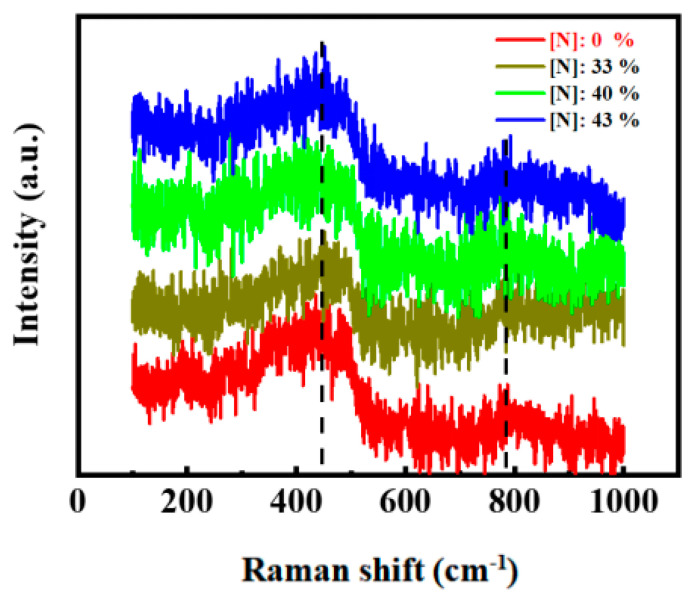
Raman spectrum of the films with different N content.

Figure 2 presents the Raman scattering spectra of the films with different N contents. A dominant Raman peak appeared at around 480 cm^−1^, which was attributed to the transverse optical vibration mode of the amorphous silicon [26]. A weak peak at ~790 cm^−1^ corresponded to the longitudinal optical vibration mode of SiC [26]. The results indicated that Si–Si and Si–C bonds existed in the form of an amorphous structure and no other Si and/or SiC nanocrystalline structure was produced in the films.

**Figure 3:**
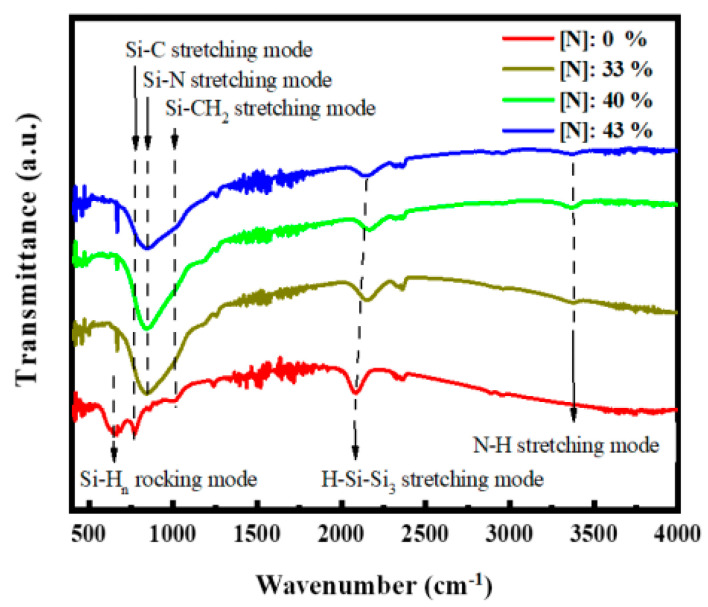
FTIR absorption spectra of the samples with different N contents.

The FTIR spectrometer was employed to clarify the bonding configurations of the films incorporated with different N contents, and the results are shown in Figure 3. The absorption peak at ~640 cm^−1^ corresponded to the SiHn rocking vibration [27]. The absorption peak at ~780 cm^−1^ corresponded to the Si–C stretching vibration [23]. The ~850 cm^−1^ absorption peak was ascribed to the Si–N stretching vibration [28]. The absorption band at ~1000 cm^−1^ was associated with the Si–CH_2_ stretching vibration, and the 1250 cm^−1^ absorption peak was ascribed to the C–H_n_ stretching vibration [27]. The peak at ∼2140 cm^−1^ bands was connected to the Si–H stretching mode [27]. The 3350 cm^−1^ peak corresponded to the N–H stretching mode [28]. As shown in Figure 3, the SiHn rocking vibration disappeared while the Si–C and Si–N stretching vibrations strengthened as the N content increased in the films. Therefore, the films mainly existed in the form of Si–C and Si–N bond structures.

**Figure 4:**
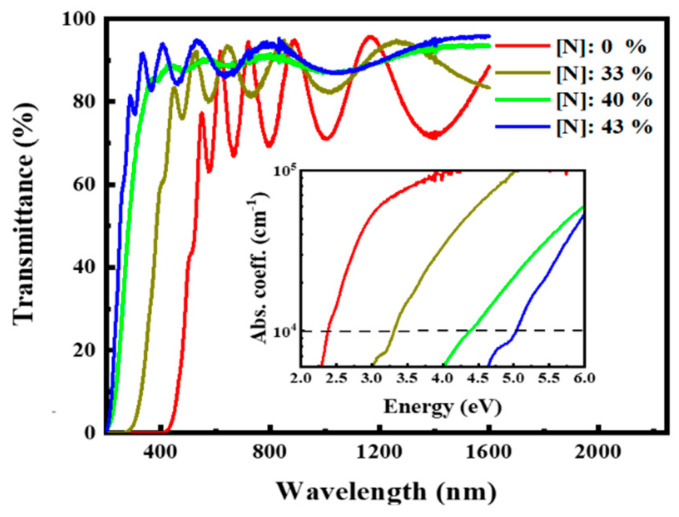
Transmission spectra of the samples with different N contents. Inset shows the corresponding absorption spectra.

Figure 4 shows the transmission spectra of the films with different N contents. With the increase in the N content from 0% to 43%, the absorption edge moved toward the short-wave direction, indicating that the increase in the N content effectively widened the optical bandgap of the films. According to the formula [29]:

(1) ad=-lnT

where *T* is the transmittance and d is the film thickness. The absorption coefficient a of the film can be obtained, as shown in the inset of Figure 4. In our case, the optical band gap E_04_ was defined as the photon energy corresponding to the absorption coefficient α = 10^4^ cm^−1^ [29]. As indicated in the inset of Figure 4, the increase in the N content from 0% to 43% resulted in a notable increase in the optical bandgap E_04_ from 2.3 eV to 5.0 eV. Our experimental results indicated that N plays an important role in the modulation of the optical bandgap of Si-rich SiCx.

**Figure 5:**
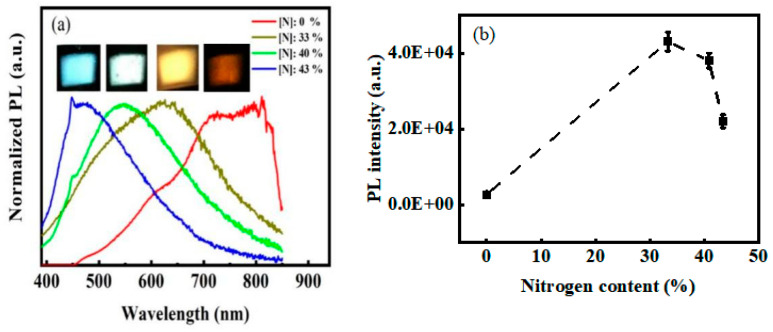
(**a**) Normalized PL spectra and (**b**) corresponding photoluminescence intensity of the films incorporated with different N content under an excitation wavelength of 325 nm.

**Figure 6:**
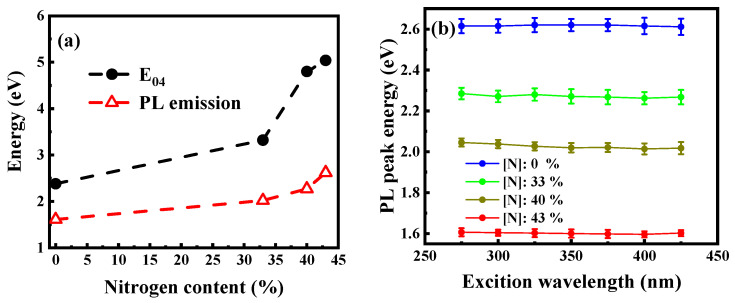
(**a**) Optical bandgap and PL peak energy of the samples as a function of N content. (**b**) PL peaks of the samples with different N contents as a function of excitation wavelengths.

Figure 5a displays the normalized PL spectra of the films incorporated with different N contents. For the films without N, the PL spectra, excited by the 325 nm line from the Xe lamp, peaked at around 800 nm. With the increase in N content from 0% to 43%, the PL peak blueshifted and gradually moved from ~800 nm to ~450 nm. All the films exhibited a strong visible light emission under 325 nm excitation that could be clearly observed by the naked eye in a bright room environment (Figure 5a). Moreover, the PL intensity increased rapidly with the increase in N content and reached the maximum when the N content was 33%. Compared with the films without N, the samples incorporated with N showed a 22-fold increase in PL intensity (Figure 5b). These results implied that N plays an important role in the PL of a-SiCx. That is, N can modulate the PL wavelength of a-SiCx and greatly increase PL intensity. Comparing Figure 4 and Figure 5b showed that the change of the optical bandgap with N content was optically consistent with the change of the PL peak energy with N content (Figure 6a). In addition, the energy of the optical bandgap was significantly greater than the corresponding PL peak energy. This result suggested that PL did not originate in band-to-band recombination. The PL spectra of the films excited at different excitation wavelengths were also studied herein to gain further insights into the PL characteristics. As shown in Figure 6b, the PL peak position barely changed with the excitation wavelength increasing from 275 nm to 425 nm. This behavior was consistent with that of defect-related PL, in which the peak position was independent of the excitation wavelength because of the narrow distribution of the defect-related localized state [30]. Thus, PL was considered to originate from defect-related luminescence centers.

**Figure 7:**
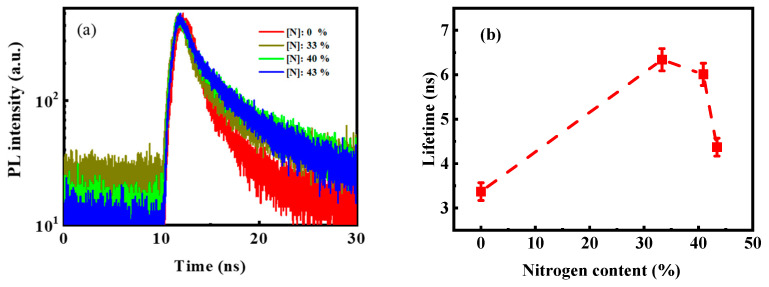
(**a**) Room-temperature luminescence decay traces and (**b**) lifetime taken from the samples incorporated with different N content, respectively.

Luminescence decay measurements were performed to further clarify the PL mechanism. Figure 7a shows the room temperature luminescence decay curves of the films. The decay curves could be well-fitted with a double exponential function. The fitted equation is as follows [31]:

(2) $$I(t)=A_{1}\exp(\frac{-t}{\tau_{1}})+A_{2}\exp(\frac{-t}{\tau_{2}})$$

where *I_0_* is the background constant, τ_1_ and τ_2_ are the luminescence lifetimes of the two decay processes, and *A_1_* and *A_2_* are the proportions of the two decay processes. According to the fitting equation, the average luminescence lifetime was calculated to be in the nanosecond range, as shown in Figure 7b. This result was consistent with the defect luminescence lifetime of Si-based materials reported in the literature [16,31]. This result further supported the speculation that PL arose from defect-related luminescence centers. By comparing Figure 5b and Figure 7b, we found that the change of the luminescence lifetime with the N content was consistent with that of the PL intensity. As shown in Figure 7b, the luminescence lifetime increased from 3.4 ns to 6.3 ns with the increase in N content from 0% to 33%. In luminescent silicon-based materials, radiative channels and nonradiative channels are involved in the recombination process of carriers. The lifetime of the nonradiative recombinations for the luminescent silicon-based materials is much shorter than that of radiative recombinations [32], and thus the increase in the measurement luminescence lifetime is mainly due to the decrease in nonradiative channels in the recombination process of carriers. Therefore, the increase in the luminescence lifetime indicated that the increase in the N content effectively passivated the nonradiative recombination centers to some extent. The Si–N bonding energy is known to be greater than the Si–Si bonding energy. Therefore, with the addition of N during the growth process, the Si–Si bond was partially replaced by the Si–N bond. The weak Si–Si bond was particularly reduced, thereby reducing the nonradiative recombination centers. The increase in the PL intensity with the N content (Figure 5) proved this view. When the N content increased from 40% to 43%, the PL intensity gradually decreased, and the luminescence lifetime decreased from 6.2 ns to 4.2 ns. This result could be attributed to the continued increase in N that aggravated the disorder of the films and, thus, led to the increase in the nonradiative recombination centers in the films. Properly modulating the N content in Si-rich SiCx could effectively widen the optical bandgap, and an efficient light emission could be obtained.

**Figure 8:**
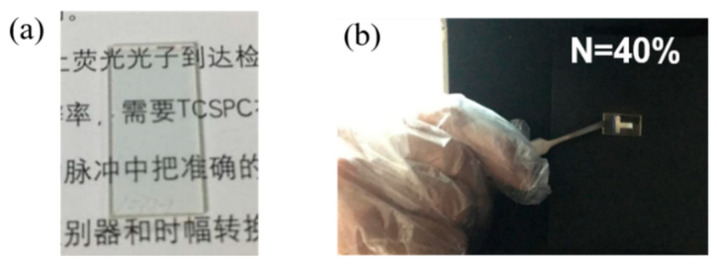
(**a**) The anti-counterfeiting symbol “T” on the quartz substrate. (**b**) The bright “T” symbol under 325 nm light irradiation.

On the basis of the above results, we further explored the application of N incorporation in the field of anti-counterfeiting. A film incorporated with 40% N was used to design the anti-counterfeiting symbol “T” on a quartz substrate. As shown in Figure 8a, the symbol “T” is transparent in the visible region as a result of the wide bandgap demonstrated in Figure 4. As expected, the quartz substrate exhibited a bright “T” symbol under 325 nm light irradiation because of its efficient PL properties, as shown in Figure 8b.

## 4. Conclusions

The effects of N incorporation on the optical properties of Si-rich a-SiCx films were investigated. The increase in N content caused a significant enhancement in PL. Relative to the samples without N incorporation, those incorporated with 33% N showed a 22-fold increase in PL. Moreover, the increase in N content blueshifted the PL from the near-infrared region to the blue region and widened the optical bandgap from 2.3 eV to 5.0 eV. As indicated by the analyses of the infrared absorption spectra and PL decay characteristics, the enhancement of PL was mainly due to the effective N passivation to the nonradiative recombination centers in the samples. Given the strong PL and wide bandgap of the N incorporated samples, they were used to design an anti-counterfeiting label. Overall, the strong tunable light emission and fast decay dynamics of the films open the possibility of applying such materials to photonics and optoelectronics integration.

## Data Availability

Data underlying the results presented in this paper are not publicly available at this time but may be obtained from the authors upon reasonable request.
